# Pyoderma gangrenosum – a review

**DOI:** 10.1186/1750-1172-2-19

**Published:** 2007-04-15

**Authors:** Uwe Wollina

**Affiliations:** 1 Department of Dermatology & Allergology, Academic Teaching Hospital Dresden-Friedrichstadt, Friedrichstrasse Dresden, Germany

## Abstract

Pyoderma gangrenosum (PG) is a rare noninfectious neutrophilic dermatosis. Clinically it starts with sterile pustules that rapidly progress and turn into painful ulcers of variable depth and size with undermined violaceous borders. The legs are most commonly affected but other parts of the skin and mucous membranes may also be involved. Course can be mild or malignant, chronic or relapsing with remarkable morbidity. In many cases PG is associated with an underlying disease, most commonly inflammatory bowel disease, rheumatic or haematological disease and malignancy. Diagnosis of PG is based on history of an underlying disease, typical clinical presentation, histopathology, and exclusion of other diseases that would lead to a similar appearance. The peak of incidence occurs between the ages of 20 to 50 years with women being more often affected than men. Aetiology has not been clearly determined yet.

The treatment of PG is a challenge. Randomized, double-blinded prospective multicenter trials for PG are not available. The best documented treatments are systemic corticosteroids and ciclosporin A. Combinations of steroids with cytotoxic drugs are used in resistant cases. The combination of steroids with sulfa drugs or immunosuppressants has been used as steroid-sparing modalities. Anti-tumor necrosis alpha therapy in Crohn's disease showed a rapid response of PG. Skin transplants and the application of bioengineered skin is useful in selected cases as a complement to the immunosuppressive treatment. Topical therapy with modern wound dressings is useful to minimize pain and the risk of secondary infections. Despite recent advances in therapy, the prognosis of PG remains unpredictable.

## Disease name

Pyoderma gangrenosum

## Definition/Diagnostic criteria

Pyoderma gangrenosum (PG) is a primarily sterile inflammatory neutrophilic dermatosis. It is characterized by recurrent cutaneous ulcerations with mucopurulent or hemorrhagic exudate. These very painful ulcers present with undermined bluish borders with surrounding erythema. In many cases, PG is associated with inflammatory bowel disease, rheumatic disorder or neoplasia [1-3].

Powell et al. [4] suggested a classification of PG into four major clinical types [see Table 1].

**Table 1:** The different clinical types of PG

**Clinical variants**	**Typical findings**
*Ulcerative PG*	Ulceration with rapidly evolving purulent wound ground
	
*Pustular PG*	Discrete pustules, sometimes self-limited, commonly associated with inflammatory bowel disease
	
*Bullous PG*	Superficial bullae with development of ulcerations
	
*Vegetative PG*	Erosions and superficial ulcers

## Epidemiology

Accurate epidemiological data on PG are missing. The peak of incidence occurs between the ages of 20 to 50 years with women being more often affected than men [2,6]. Cases in infants and adolescents account for only 4% of PG. PG in elderly people has occasionally been reported [7]. The general incidence has been estimated to be between 3 and 10 per million per year [8].

## Clinical presentation

**Figure 1:**
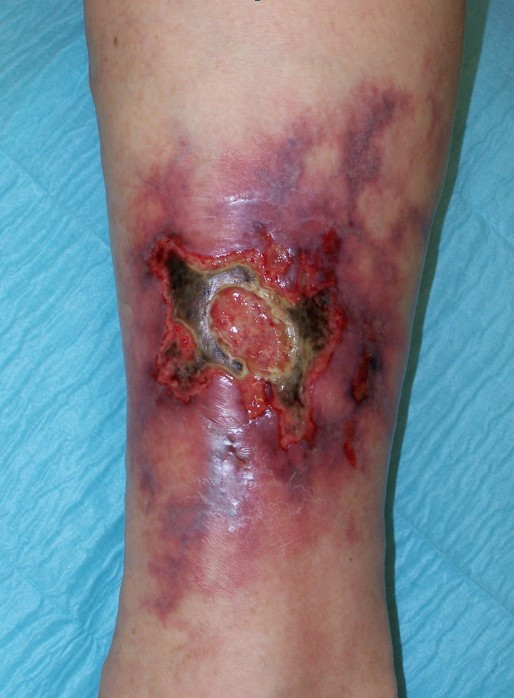
Acute rapid growing pyoderma gangraenosum with undermined violaceous borders.

**Figure 2:**
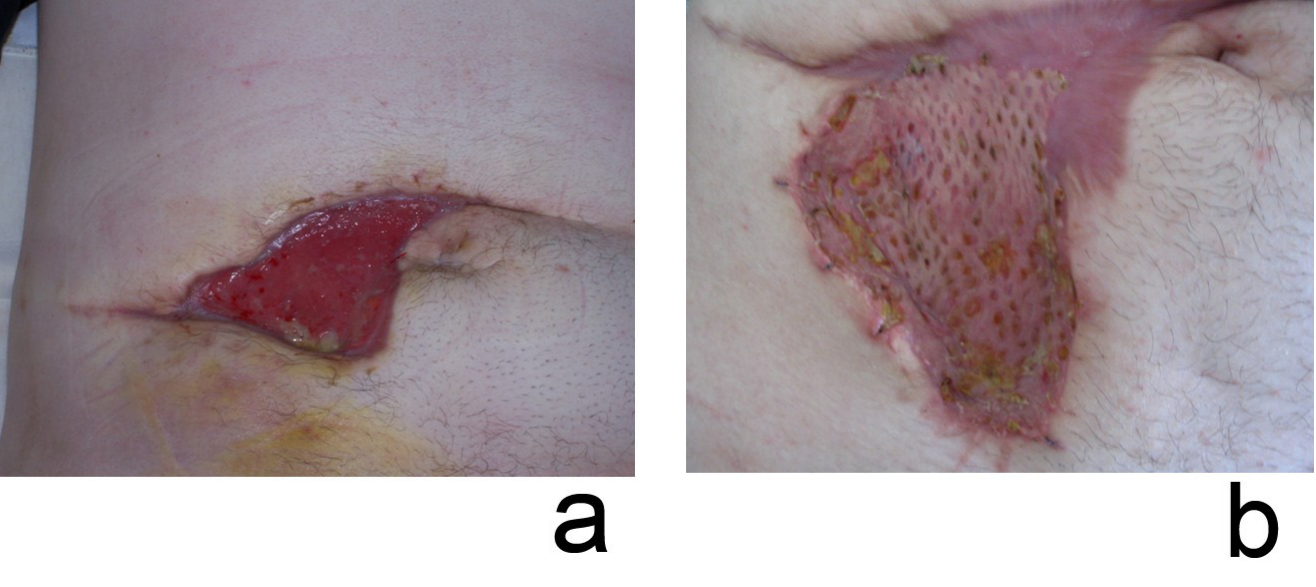
Postsurgical pyoderma gangraenosusm [a] before treatment; [b] after immunosuppressive treatment with oral prednisolone and mesh-graft transplantation.

**Figure 3:**
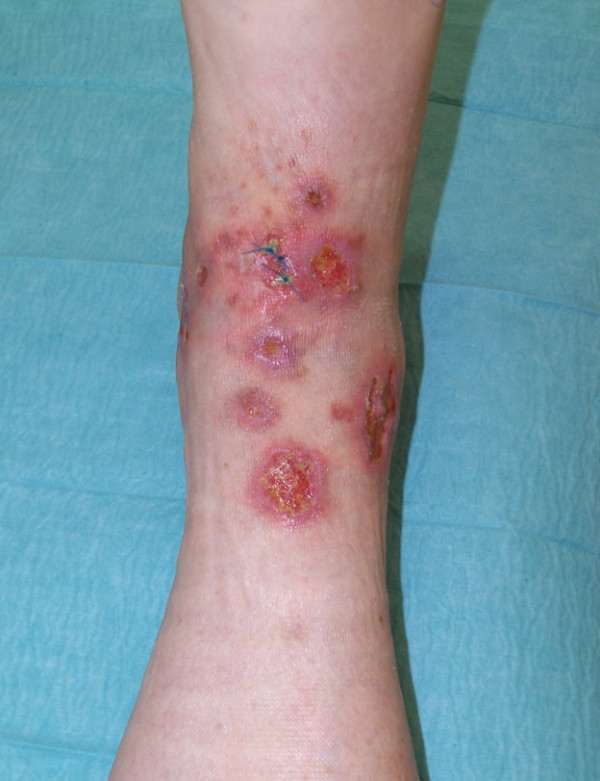
Ecthyma-like pyoderma gangraenosum.

**Figure 4:**
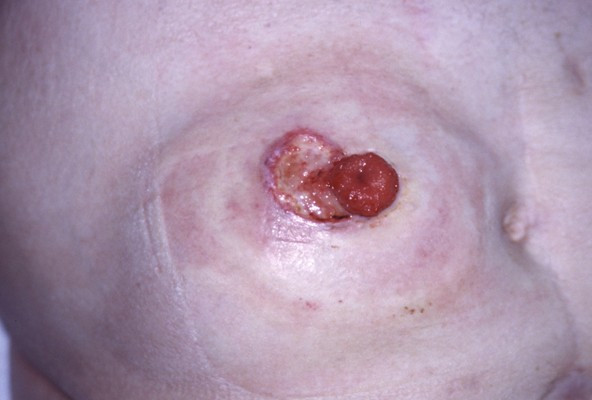
Peristomal pyoderma gangraenosum in ulcerative colitis.

PG occurs most commonly on the lower legs with preference for the pretibial area [Fig. 1 &2]. PG has been reported on other sites of the body as well, including breast, hand, trunk, head and neck, and peristomal skin [Fig. 3 &4]. Extracutaneous manifestations include involvement of upper airway mucosa, eye [9-12], genital mucosa [13], sterile pulmonary neutrophilic infiltrates [14] or spleen infiltrates [15], and neutrophilic myositis [16]. Sterile cortical osteolysis has been observed adjacent to PG ulcers as another extracutaneous manifestation of the disease [17].

The development of PG is a feared complication after breast surgery and other surgical procedures [18-20]. It seems to me that areas rich in subcutaneous fat tissue bear a somewhat higher risk than others but systematic investigations in this field are missing.

The ulcer starts as a follicular pustule with rapid growth, tissue necrosis and enlargement of the area. The surrounding skin is erythematous with infiltration end oedema. The ulcer borders are typically undermined and violaceous or bluish [Fig. 1]. The ulcers develop a purulent cover, which rapidly becomes malodorous due to secondary infection. A strong sensation of pain often is associated to PG [1,2,10].

In larger series, about 50% of patients show an underlying disorder. Ulcerative colitis is found in 10–15% of cases. Another associated disease is Crohn's regional enteritis with a frequency close to that of ulcerative colitis [2,21,22]. On the other hand, less than 3% of patients with Crohn's disease or ulcerative colitis develop PG [23,24]. Hepatitis C, seronegative rheumatoid arthritis, spondylitis, and a broad spectrum of lymphoproliferative disorders including monoclonal gammopathies, leukaemia, lymphoma, and myelodysplastic syndrome have been described in association with PG [2,4,25,26].

The PG-ulcers associated with arthritis seem to have a poorer prognosis than others. In a study covering 2 years 78.9% of PG-ulcers in general healed versus only 23.4% in arthritis-associated PG [27]. In such cases PAPA syndrome has to be considered [28]. PG involvement of the hands shows a higher percentage of lymphoproliferative than chronic inflammatory bowel disease [29].

PG is a typical clinical presentation of the PAPA syndrome also known as familial recurrent arthritis [28]. The autosomal dominant inherited disease is characterized by nonaxial destructive arthritis, severe cystic acne and PC. PAPA syndrome is caused by mutations in the PSTPIP1 gene on chromosome 15 involved in regulation of inflammatory response [30].

PG can arise as a consequence of drug therapies. Recent examples of drug-induced PG include compounds such as propylthiouracil [31], pegfilgastrim – a granulocyte-stimulating factor [32], and gefinib – an inhibitor of epidermal growth factor receptor [33].

## Etiology and pathogenesis

PG was initially thought to be caused by bacterial infection in the immunocompromised host [1]. Fulbright *et al*. [20] hypothesized that PG results from an aberrant immune response to yet unidentified factors. Depositions of proteins in skin vessels in PG lesions have suggested an Arthus-like reaction [34]. Since inflammatory bowel disease is the most common underlying disorder, cross-reacting antigens in the bowel and the skin could be responsible for secondary cutaneous manifestation [2].

Cellular analysis in PG demonstrated aberrant integrin oscillations on neutrophils and aberrant neutrophil tracking of patients with PG [35,36]. Pathways to protect the epidermis from neutrophil infiltration seem to be insufficient in PG resulting in tissue necrosis.

## Diagnostic methods

Diagnosis of PG relies on clinical signs first and is supported by biopsy for histopathology. Knowledge of the patient's history for possible underlying disease and specific investigations based on that background are necessary. Therefore diagnosis is made by exclusion of other possible disorders. No laboratory parameter for PG is available. The histopathology of PG is nonspecific and changes with the stage of lesion. The initial lesions show a deep suppurative folliculitis with dense neutrophilic infiltrate. In about 40% of cases, leukocytoclastic vasculitis is present. PG with [necrotizing] granulomatous inflammation has been described [3,34,37]. These reports illustrate the difficulties of a diagnosis based solely on histopathology since concomitant occurrence of PG and systemic necrotizing vasculitis has been observed.

## Differential diagnosis

Six disease categories may imitate the clinical appearance of PG [38]:

(a) Vascular occulusive or venous disease including calciphylaxis which is particular painful and rapidly evolving.

(b) Vasculitis. That is a particular challenge when PG develops in a patient with vasculitic rheumatoid arthritis. Differential diagnoses of this topic include Wegener's disease, phospholipid syndrome, or in Adamantiades-Behcet's disease etc.

(c) Cancer. Again the differential diagnosis is most complicated in patients with lymphomas or leukaemia. Where the specific cutaneous lesions may present as suppurative ulcers.

(d) Infectious disease. Ecthymata and deep [tropical] mycoses like sporotrichosis may resemble PG. The rapid onset of post-surgical PG often reminds of acute deep skin infection such as erysipelas or gangrene [18]. Late syphilis has become more common again and may develop suppurative ulcerations. Deep viral herpetic infections can resemble PG.

(e) Exogenous tissue injury. Facticious panniculitis as a part of the Münchhausen syndrome may masquerading as PG [39]. Insect or spider bites can cause necrotizing skin ulcers.

(f) Drug reactions. Pustular drug reactions may masquerade as pustular PG.

## Treatment

### Systemic therapy

For patients with a more widespread disease or rapidly progressive course, systemic treatment is mandatory. Corticosteroids, like prednisolone, 1 to 2 mg per kg per day, are widely used for initial therapy [2,4,25]. Trying an initial high-dose therapy aims to prevent progression and rapidly stop inflammation. Action of pulse therapy with suprapharmacological doses of corticosteroids [1 g of methylprednisolone] is faster [40]. Since this treatment may cause fatal side effects in patients with cardiovascular disease on diuretics, patients have to be carefully selected.

Ciclosporin A is an inhibitor of T-lymphocyte activation. Immunosuppressive therapy with ciclosporin A has become an accepted treatment for widespread PG after initial steroids or in combination with steroids. In many cases, steroids can be completely be replaced by ciclosporin A [41,42]. Doses of 2 to 3 mg ciclosporin A/kg body weight and day show efficacy in PG [43]. During therapy with ciclosporin A it is necessary to control blood pressure and creatinine. The drug induces an early response but has no impact on the incidence of recurrences [44]. Therefore, combination with other drugs can become necessary even after initial response to ciclosporine A monotherapy.

Sulfa drugs are useful in milder cases of PG. The combination of steroids with diaminodiphenylsulfone [dapsone] up to 200 mg daily [only for patients with a normal glucose-6-phosphate dehydrogenase level] is the most popular. Dapsone inhibits neutrophil migration and production of reactive oxygen species and exerts a variety of other anti-inflammatory activities [4,45]. Formation of met-hemoglobin needs regular monitoring during this treatment.

Clofazimine is a scavenger of hypochlorus acid, thus reducing the chlorination of proteins by neutrophils. It stimulates phagocytosis and superoxide production and has a direct antibacterial activity as well. In my opinion the efficacy of clofazimine 300 to 400 mg/day is comparable to that of sulfa drugs as dapsone. A common side effect is hyperpigmentation [46].

Thalidomide shows immunomodulatory activity such as suppression of tumour necrosis factor-alpha, basic fibroblast growth factor and neutrophil chemotaxis. It was used in combination with steroids [47]. Thalidomide is teratogenic and therefore absolutely contraindicated in fertile women.

Colchicine, an inhibitor of the spindle-apparatus with anti-inflammatory properties, was employed in two patients with PG from Greece [48]. It may be used as a single agent or in combination with prednisolone depending on the severity of disease. Gastrointestinal adverse effects may limit its use.

Azathioprine [100–150 mg/day] is a cytotoxic drug usually administered for its steroid-sparing effects. Its action is delayed of at least about 2 to 4 weeks. Blood counts and transaminases should be monitored [2,4,45]. There is a wide inter-individual variability in azathioprine metabolism due to a common genetic polymorphism of thiopurine methyltransferase, a major metabolizing enzyme of azathioprine. Azathioprine and sulfasalazine are a good choice when the underlying disease is ulcerative colitis or Crohn's disease.

Another cytotoxic drug that has been used in PG is cylophosphamide. Cyclophosphamide pulse therapy followed by either azathioprine or methotrexate is an effective treatment in steroid-refractory Crohn's disease. In cases of Crohn's associated PG may achieve long term remissions with this regimen lasting for up to 30 months [49].

Mycophenolate mofetil is an inhibitor of the purin synthesis selectively reducing the proliferation of T- and B-lymphocytes. With the dosage of 2 g/day there is a very low risk of renal and hepatic side effects. Mycophenalate mofetil was used in peristomal PG and other refractory cases [50,51]. Recently, a case of staphylococcal and pseudomonal sepsis has been reported in a mycophenolate mofetil treated PG-patient [52].

Enteric-coated mycophenolate sodium [ECMPS] is an advanced formulation delivering mycophenolic acid. At a dosage of 720 mg ECMPS exhibits equivalent mycophenolate acid exposure to mycophenolate mofetil 1000 mg. Recent trials demonstrated that ECMPS has a similar safety profile than mycoephenolate mofetil [53].

Tumour necrosis alpha inhibitor infliximab was reported to be effective in PG associated with inflammatory bowel disease at a dosage of 5 mg/kg body weight. Infliximab is given by infusion at weeks 0, 2 and 6, and every 8 weeks thereafter. The chimeric antibody is usually combined with low-dose methotrexate for Crohn's disease [54,55]. In a small series including 4 patients with fistulating Crohn's disease and PG infliximab was given either as a single infusion or a series of three infusions at a dosage of 5 mg per kg body weight/day. All patients demonstrated a rapid healing of PG within 4 weeks after starting the treatment. Healing was complete and relapses were not observed [56]. In another study four patients with PG-ulcers and Crohn's disease showed a marked improvement after a single infusion of infliximab [57]. Since infusion reactions can occur in 3% to 17% of patients with Crohn's disease that are antibody associated the concurrent administration of steroids and the use of immunosuppressant like methotrexate or azathioprine has been recommended [58].

The positive effect on PG has also seen with another tumour-necrosis factor alpha antagonist etanercept, a fusion protein [59]. Etanercept is applied by subcutaneous injections of 50 mg twice weekly. Because there is a risk of reactivation of tuberculosis during anti-tumour necrosis alpha therapy patients have to be screened for tuberculosis before and during treatment [58].

Tacrolimus, a selective calcineurin inhibitor, was used at low dosages of 0.1 mg per kg body weight/day. The treatment was monitored to maintain a serum level of between 4 and 6 ng/L. Complete clearance of skin lesions was observed in two patients with underlying ulcerative colitis [60].

Since PG is a neutrophilic disease, removal of activated neutrophiles should improve the symptoms. Leukocytapheresis, where white blood cells are removed extracorporeal, and granulocyte adsorptive apheresis, a more selective procedure, have been used in single cases with success. These methods have been used in single cases unresponsive to systemic standard therapy with success [61,62].

### Topical therapy

Topical treatment is important in any case when ulcers have developed. Moist wound management is a cornerstone of wound management. Since most ulcers show heavy exudates, foam dressings or laminate dressings composed of different layers are recommended. In case of sloughy or purulent covered lesions, semiocclusive dressings are contraindicated. In such cases, wet compresses with sterile saline solution or Ringer-lactate solution and alginate dressings are useful. Pain relief and improvement of odor have also been observed with compresses [25].

For small flat lesions without secondary infection, topical high potent corticosteroids are in use. They are rarely capable of inducing remissions except in peristomal PG [4,10,11]. Topical calcineurin inhibitors like tacrolimus or pimecrolimus were used in some cases with success [4,63,64]. Dramatic improvement has been observed in particular in peristomal PG [65,66].

### Surgical therapy

Surgery has to be used with caution since it can be trigger PG [19]. Any surgical procedure has to be done as an adjunct to immunosuppression only in patients with stable disease or partial remission. Autologous split-skin grafts have been used with variable outcome [[67] Fig. 3]. A significant disadvantage of split-skin grafts is the necessity to create a new wound at the donor site. New developments include the use of bioengineered skin, like the dermal regeneration template Integra^®^, hair follicle stem cell-derived autologous keratinocyte sheets Epidex^® ^or hyaluronic acid-based autologous keratinocyte delivery system Laserskin^® ^[18,25,50,68-70]

## Prognosis

**Figure 5:**
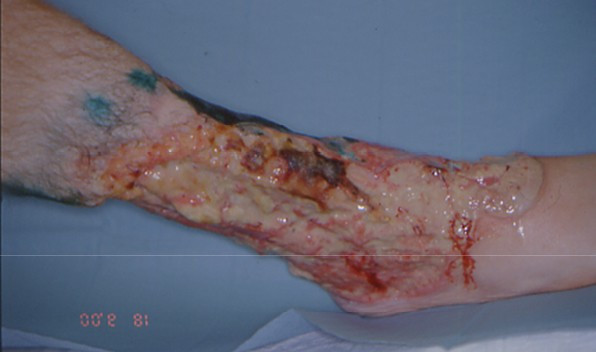
Deep purulent ulcerations due to pyoderma gangraenosum. In this patient later on a lower limb amputation was necessary, since he did not response to drug therapy.

Despite advances in management, the long term outcome of PG remains unpredictable. We have seen one young patient who did not respond to intense drug therapy anymore. There fore because of intractable apin and toxicity of the large wound a lower limb amputation was performed eventually [Fig. 5]. Even in those patients who respond to drug therapy relapses occur. They are seen in about 70% of patients treated with prednisolone and 66% of patients treated ciclosporine A [44]. In cases where long term treatment is justified a careful monitoring of possible adverse effects is mandatory [5,25]. In those cases with a complete clinical remission the internal treatment dosage should be tapered down. PG is still a potentially life-threatening with a mortality rate of up to 30% in some series [70].

## Open questions

Our knowledge about pathogenesis and individual risk factors for the development of PG is still rather limited. This hampers the efforts for prevention. In the treatment of PG controlled, prospective and randomized trials have yet not been established. Such an approach would be possible only in a multinational trial because of the rareness of this disease. It would further been limited by the various clinical types and courses of this disease. A major point for further investigations is the calculation of prognosis. The course of this disease is unpredictable and the manifestations range from very limited and superficial ulcerations to widespread disease with extracutaneous manifestations and life threatening course. The new attempts of treatment with biologics needs careful investigation since this could provide a new understanding of the disease.
